# Listerial rhombencephalitis diagnosed by repeat blood culture despite negative CSF PCR: a case report

**DOI:** 10.1128/asmcr.00063-26

**Published:** 2026-07-23

**Authors:** Simona Arientová, Radmila Galušková, Lenka Ceprová, Michal Jareš, Michal Holub

**Affiliations:** 1 Department of Infectious Diseases, Military University Hospital Prague 48235, Prague, Czech Republic; 2 Department of Neurology, Military University Hospital Prague 48235, Prague, Czech Republic; 3 Department of Radiology, Military University Hospital Prague 48235, Prague, Czech Republic; Vanderbilt University Medical Center, Nashville, Tennessee, USA

**Keywords:** case report, rhombencephalitis, *Listeria monocytogenes*, cerebrospinal fluid, PCR, blood culture

## Abstract

**Background:**

Rhombencephalitis is a rare inflammatory disorder of the brainstem and cerebellum, most commonly associated with autoimmune, paraneoplastic, or vascular etiologies. *Listeria monocytogenes* is an uncommon but potentially severe infectious cause, and diagnosis may be challenging due to the limited sensitivity of cerebrospinal fluid testing.

**Case Summary:**

An adult patient presented with subacute, progressive brainstem symptoms. Neurological examination and magnetic resonance imaging findings were consistent with rhombencephalitis. Cerebrospinal fluid analysis demonstrated inflammatory changes; however, PCR testing for *L. monocytogenes* was negative. Additional cerebrospinal fluid findings included moderately elevated interleukin-6 and chemokine CXCL13 levels and detectable Epstein–Barr virus DNA. Blood cultures subsequently yielded *L. monocytogenes*, confirming the diagnosis. Targeted antimicrobial therapy was initiated, and adjunctive corticosteroids were administered because of severe neurological involvement. The patient showed clinical stabilization and neurological improvement during follow-up.

**Conclusion:**

This case demonstrates that listerial rhombencephalitis may occur even with negative cerebrospinal fluid PCR results and highlights the importance of empiric therapy covering *Listeria* until an alternative etiology is confirmed. Marked inflammatory and immunological cerebrospinal fluid abnormalities may accompany severe disease and should prompt careful clinical assessment.

## INTRODUCTION

Central nervous system (CNS) infections caused by *Listeria monocytogenes* are associated with high mortality rates, ranging from 17% to 48%, among the highest reported for bacterial meningitis and comparable to those of pneumococcal meningitis (1). Clinical outcomes depend on host factors, timeliness of antimicrobial therapy, and the pattern of CNS involvement. CNS listeriosis primarily affects neonates, older adults, and individuals with impaired cell-mediated immunity, including people living with HIV (2). However, rhombencephalitis, a rare brainstem manifestation of neurolisteriosis, frequently occurs in otherwise healthy adults, with nearly 70% of reported patients being immunocompetent (3). We report a case of listerial rhombencephalitis in an immunocompetent patient with repeatedly negative cerebrospinal fluid (CSF) PCR results and delayed microbiologic confirmation, highlighting the diagnostic importance of compatible clinical and radiologic findings despite the absence of early laboratory confirmation.

## CASE PRESENTATION

A previously healthy 61-year-old gardener sustained a facial injury 4 days before admission while chopping wood, during which wood splinters and leaf debris entered his oral cavity. On the same day, he developed fever (up to 38°C), chills, and rigors. One day before admission, symptoms recurred and were accompanied by vomiting and several episodes of collapse. At presentation to the Emergency Department, he reported neck stiffness, persistent headache, and right-sided facial paresthesia and was admitted to the Neurology Department of a regional hospital. Brain computed tomography (CT), performed on hospital day 2, showed no acute abnormalities. CSF analysis was interpreted as aseptic meningitis (Table 1). Empiric treatment with intravenous ceftriaxone (2 g/day) and acyclovir (750 mg three times/day) was initiated, as neuroborreliosis is relatively common in this region. Owing to the absence of an infectious diseases service, the patient was transferred on hospital day 3 to our Department of Infectious Diseases.

**TABLE 1:** Abnormal cerebrospinal fluid findings

Parameter	Value	Reference range
Leukocytes (cells/mL)^*a*^	890	0–5
Protein (g/L)	1.28	0.18–0.43
Lactate (mmol/L)	2.23	1–2
Interleukin-6 (pg/mL)	>1,000	<7.9
CXCL13 (pg/mL)^*b*^	469.58	<20
EBV DNA (copies/mL)^*c*^	1,033	200

^*a*^ Granulocytic pleocytosis.

^*b*^ B-cell chemoattractant.

^*c*^ Epstein–Barr virus viral load.

**Fig 1:**
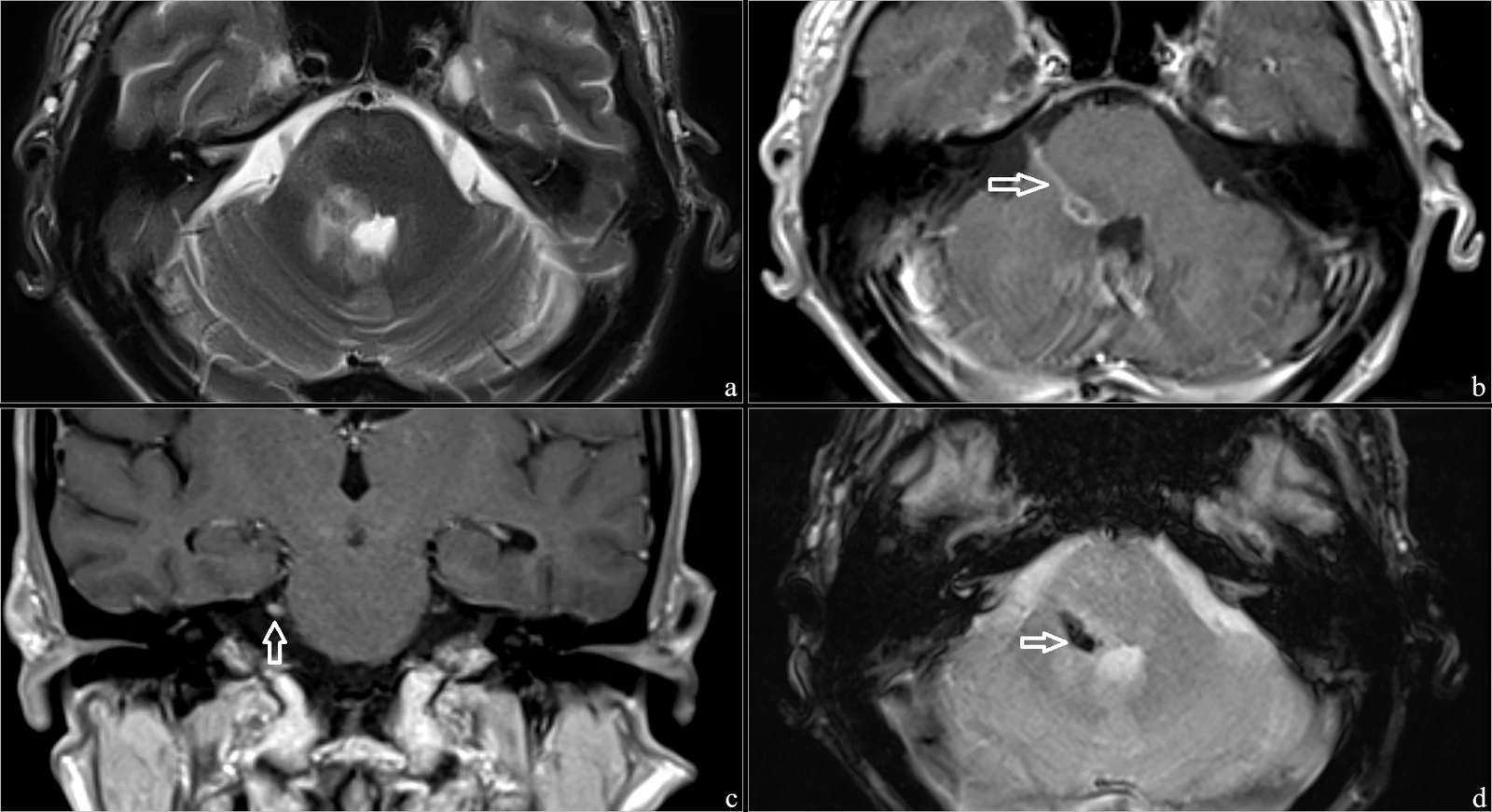
(**a**) T2-weighted sequence in the axial plane showing signal abnormalities in the right portion of the brainstem, specifically involving the lateral mixed system. (**b**) T1-weighted fat-suppressed contrast-enhanced sequence in the axial plane. (**c**) T1-weighted fat-suppressed contrast-enhanced sequence in the coronal plane both demonstrating significant involvement of the trigeminal nerve with possible involvement of its nuclei. (**d**) T2-weighted gradient-echo sequence in the axial plane suggesting a small hemorrhage extending from the pons to the right cerebellar peduncle at the midbrain–pons junction.

CSF PCR testing using the BioFire FilmArray Meningitis/Encephalitis Panel, available on the second day after transfer, was negative for all 14 viral and bacterial pathogens included in the panel. Additional PCR assays for *Mycobacterium tuberculosis* and *L. monocytogenes*, as well as for fungal pathogens including *Aspergillus* spp., *Candida albicans*, *Candida glabrata*, *Candida krusei*, and *Cryptococcus neoformans*, were also negative. Epstein–Barr virus (EBV) DNA was the only positive CSF PCR finding. Based on these findings, acyclovir was discontinued, and ampicillin was not initiated. Despite ongoing ceftriaxone use, the patient’s neurologic condition progressively deteriorated, with worsening nuchal rigidity, persistent right-sided facial dysesthesia, and newly developed nystagmus and dysphagia. Brain magnetic resonance imaging (MRI) demonstrated rhombencephalitis (Fig. 1a through d). Because autoimmune rhombencephalitis was suspected, intravenous methylprednisolone (1 g/day) was administered for 2 days. Serologic testing for Lyme borreliosis, HIV, syphilis, and tick-borne encephalitis was negative, as were CSF PCR assays for *Leptospira interrogans* and *Toxoplasma gondii*. Ceftriaxone was therefore discontinued, and the patient was transferred to the Neurology Department.

During the subsequent hospitalization, dysphagia worsened and required placement of a percutaneous endoscopic gastrostomy tube. MRI findings were interpreted as potentially vascular in origin; however, cerebral angiography was normal. Extensive testing for autoimmune and paraneoplastic etiologies was unrevealing, including serum and CSF analyses for antibodies against N-methyl-D-aspartate receptor, α-amino-3-hydroxy-5-methyl-4-isoxazolepropionic acid receptor, γ-aminobutyric acid B receptor, voltage-gated potassium channel complex, dipeptidyl-peptidase-like protein 6, and multiple onconeural antibodies. Autoimmune disease was further excluded by negative isoelectric focusing for immunoglobulins and free light chains. On the third day of hospitalization in the neurology department, the patient again became febrile (38.6°C), and repeat blood cultures were obtained. On the fifth hospital day, one of six blood culture bottles yielded *L. monocytogenes*. Identification was confirmed by BioFire FilmArray, MALDI-TOF mass spectrometry, and conventional culture methods. The isolate was classified as serotype 4b by immunologic serotyping and PCR-based serogrouping.

**Fig 2:**
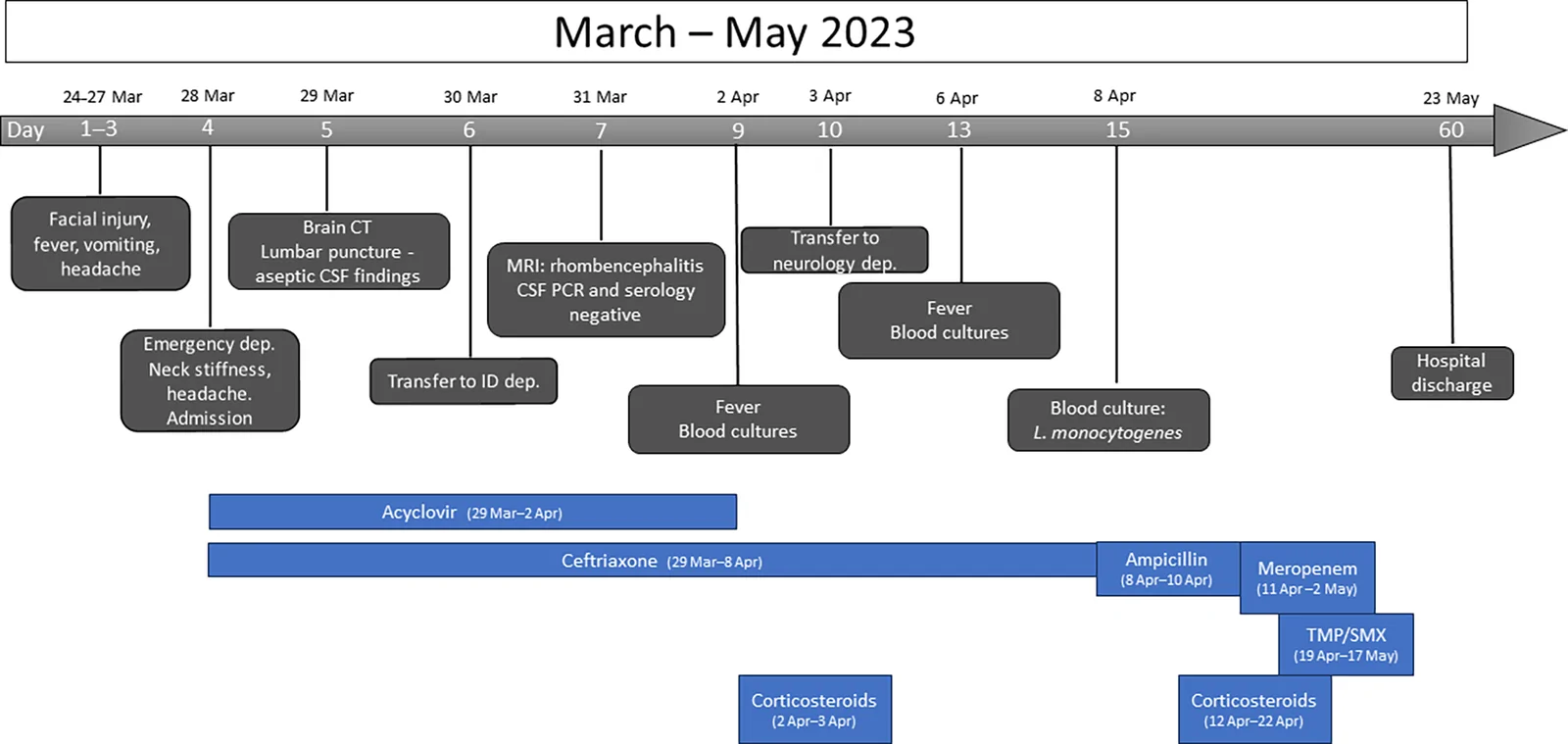
Timeline of key events during hospitalization and follow-up.

Intravenous ampicillin (12 g/day) was initiated immediately after microbiologic confirmation. Because of suspected nosocomial respiratory infection with marked bronchial secretions and crackles on auscultation, antimicrobial therapy was subsequently escalated to intravenous meropenem (6 g/day), which was continued for 3 weeks. Oral trimethoprim–sulfamethoxazole (TMP-SMX; 960 mg four times/day) was added on hospital day 15 and continued for 4 weeks. Adjunctive methylprednisolone (1 g/day for 4 days) was also administered. The overall timeline of hospitalization is shown in Fig. 2.

### Follow-up and outcomes

More than 1 year after hospitalization, the patient continued to exhibit persistent left-sided hemiparesis and hemiataxia, together with oculomotor dysfunction and visual field deficits without a clear MRI correlate. At the last follow-up (autumn 2025), he was ambulatory with a walker, while dysphonia and dysphagia had resolved.

## DISCUSSION

The diagnosis of CNS listeriosis traditionally depends on CSF culture; however, its sensitivity remains limited, increasing only from approximately 33% to 41% even with repeated cultures (4). PCR has therefore become the preferred diagnostic modality; however, false-negative PCR results may occur, particularly when low CSF volumes are analyzed (5). In our patient, CSF PCR testing for *L. monocytogenes* remained negative despite being performed according to standard protocols using a commercial real-time PCR assay capable of detecting genotype 4b strains. Ultimately, the diagnosis was established only after delayed blood culture positivity, with growth detected in just one of six culture bottles. Similar observations have been reported in another case of listerial rhombencephalitis in which repeated CSF PCR testing remained negative and diagnosis was achieved only through plasma cell-free DNA testing (6). Together with the isolation of *L. monocytogenes* from only one of six blood culture bottles in our patient, these findings suggest that bacterial burden in both CSF and blood may be low in some cases of neurolisteriosis. Importantly, these observations underscore that negative CSF PCR results do not exclude neurolisteriosis and that empiric antimicrobial therapy active against *L. monocytogenes*, particularly ampicillin, should be initiated or maintained whenever compatible clinical and radiologic findings raise suspicion for listerial rhombencephalitis.

The clinical presentation in our patient also supports the hypothesis of intra-axonal CNS spread via cranial nerves. Early unilateral facial dysesthesia suggested trigeminal nerve involvement, a finding previously described in patients with listerial rhombencephalitis (7). Unlike previously reported cases, however, our patient had no preceding gastroenteritis but instead experienced direct oral exposure to wood splinters and plant debris immediately before symptom onset. To our knowledge, direct oral inoculation preceding neurolisteriosis has not previously been reported. This mechanism may also explain the unusually short interval between exposure and neurologic manifestations compared with the longer latency typically proposed for vagal spread from the gastrointestinal tract (8).

The antimicrobial strategy in this case was complex. Meropenem was continued longer than would usually be recommended for nosocomial infection management, and TMP-SMX was added because of concerns regarding the potentially suboptimal activity of meropenem against *L. monocytogenes* and the favorable CNS penetration of TMP-SMX. Although the patient ultimately improved, earlier de-escalation to narrower-spectrum therapy would likely have been more consistent with antimicrobial stewardship principles (9).

The role of adjunctive corticosteroids in neurolisteriosis remains controversial. Current recommendations generally advise discontinuation of corticosteroids once *Listeria* infection is confirmed, partly based on the French MONALISA study, which demonstrated increased mortality among patients receiving corticosteroids (10). In contrast, a Dutch cohort suggested a possible survival benefit associated with adjunctive dexamethasone (11). In our patient, corticosteroids were initially administered because autoimmune rhombencephalitis was suspected and were later continued adjunctively after diagnosis had been established. Whether this contributed to recovery remains uncertain.

Finally, low-level EBV DNA detected in CSF likely represented reactivation within recruited B cells during blood–brain barrier disruption rather than primary CNS infection. Elevated CSF CXCL13 and interleukin-6 levels supported active neuroinflammation and intrathecal immune activation, findings previously described in neuroinfectious diseases such as neuroborreliosis and neurosyphilis (12, 13).

### Conclusion

This case emphasizes the diagnostic challenges of *Listeria* rhombencephalitis, reminding clinicians to consider *Listeria* as a potential cause of rhombencephalitis, even in immunocompetent patients with negative PCR results.

## Data Availability

All data supporting the findings of this case report are included in this article.
